# A web of gaps: a discussion of research strands concerning Global South families with a disabled child

**DOI:** 10.1080/16549716.2017.1337355

**Published:** 2017-06-26

**Authors:** Xanthe Hunt, Brian Watermeyer

**Affiliations:** ^a^ Department of Psychology, Stellenbosch University, Stellenbosch, South Africa; ^b^ Department of Rehabilitation Science, University of Cape Town, Cape Town, South Africa

**Keywords:** Disability, low- and middle-income countries, poverty, Amartya Sen

## Abstract

**Background:** In low- and middle-income countries (LMICs), limited access to a range of supports means that families often carry primary responsibility for the care of a disabled child. The impact of this responsibility is poorly understood.

**Objective:** To present a selective review, critique, and comparison of the prominent areas of research aimed at understanding families with disabled children in the Global South.

**Design:** We compare and critically discuss prominent bodies of literature concerning the family-disability-poverty nexus in LMICs.

**Results:** Three prominent bodies of literature concerned with families with a disabled child in LMICs are reviewed. These were selected based on their relative prevalence in a large review of the literature, and comprise (1) work concerning quality of life (FQOL) of families with a disabled child; (2) interventions aimed at supporting families with a disabled child in LMICs; and (3) the ways in which culture mediates the families’ experience of disability. FQOL research points to poverty as a primary source of family distress, and directs our focus towards families’ own expertise in coping with their circumstances. Intervention literature from LMICs highlights the family as the unit of analysis and praxis concerning disabled children, and reminds us of the contextual factors which must be considered when working with their families.

**Conclusions:** Culturally oriented research on poverty, disability, and the family nuances our understanding of the locally-determined priorities of families with a disabled child in LMICs. All three research strands carry benefits, limitations and gaps. The complexity of understanding families with a disabled child in LMICs comes to the fore, directing us away from narrow application of any single theoretical or research framework. Future researchers may draw on insights provided here in creating a more integrated approach.

## Background

In low- and middle-income countries (LMICs), limited- or non-access to a range of social supports means that families often carry primary responsibility for the care of disabled children. The impact of this responsibility, in contexts of poverty, is complex and poorly understood. Several research strands have investigated the circumstances of families with a disabled child, within a variety of domains (Note: the manner in which we use the term families here incorporates a range of ways of being in which people who are related by marriage or blood find themselves arranged). However, each of these strands of work elides certain realities of such families in the Global South (LMICs), despite making some useful contributions.

The stimulus for the paper was a research project currently being conducted by BW, involving the collection of in-depth interview data on the experiences of families with disabled children living in poor communities around Cape Town, South Africa. An examination of the literature in support of the project uncovered the overall paucity and limited applicability of existing research in the area relevant to LMIC contexts.

This paper, therefore, presents a selective review, critique, and comparison of the prominent areas of research aimed at understanding families with disabled children in the Global South. Reading these bodies of work against one another, and in light of theoretical work on poverty, we aim to reflect on what this process teaches us, and to provide key insights by which we can set priorities for research in the field in LMICs. In the sections which follow, we provide a concise theoretical orientation, dealing with current ideas on the nature and assessment of poverty, and the interrelationship between poverty and disability. The conceptual framework on poverty described here is especially flexible, making it useful for understanding diverse contexts. It forms a backdrop against which we consider the research material which is the topic of this paper.

## Conceptualizing poverty, and its relationship to disability

Teasing out disadvantage based upon disability from that caused by poverty is especially difficult in settings where poverty is endemic. Disability in the family may tax resources in subtle ways, reducing household labour power or well-being, or leading to additional expenses such as transportation to medical facilities.

Theoretical propositions relevant to our discussion appear here on two levels. The first comprises ideas on how poverty is imagined, and the second addresses the relationship between disability and poverty.

The past twenty years has brought an important shift in how poverty is conceptualised and evaluated in development economics – a change from poverty defined by straightforward income or consumption levels, to a view concerned with access to a range of essential resources (a multidimensional approach) [1]. Central among these frameworks is the work of Amartya Sen. Sen [2–5] pioneered the so-called ‘capabilities approach’ which assesses poverty in terms of a standard of living described by the capability to conduct various ‘functionings’ central to human flourishing. ‘Development’, within this framework, is believed to have happened on attainment of the freedom to engage in meaningful activities. Besides the achievement of desirable states such as being well-nourished and sheltered, such functionings include the exercise of freedom of movement, or the ability to form and maintain a family. The approach focusses on what one is able to do and be in one’s society, rather than on simple accumulation. This has particular relevance for thinking about the lives of people with disabilities, and their families, whose circumstances may present complex, uneven patterns of deprivation.

A large store of literature attests to the notion that disability and poverty share a bi-directional relationship, as part of a so-called ‘vicious cycle’ [6–10]. It argues that people living in poverty are more vulnerable to disability, and that disability, in turn, may lead to a descent into, or a cementing of, poverty. Recent research has revealed this position to be an oversimplification. Further, the disability-poverty relationship in the Global South appears more complex than in wealthier nations, as here poverty is often the norm rather than the exception [7,11–13]. Nevertheless, it seems reliable that, in LMIC contexts: (1) the family unit as a whole is poorer when one member has a disability (and this poverty may be intergenerationally transmitted); (2) disabled family members are more affected by household poverty than others; and (3) community-wide economic improvement may not reach disabled individuals and their families [14].

The limited empirical accounts that we have of the real-life economic implications of disability for families are illuminated by Sen’s capabilities model. Indeed, the implications of Sen’s work for disability more generally are readily evident [15]. In terms of the influential ‘social model’ view [16], disability is defined as ‘the loss or limitation of opportunities that prevents people who have impairments from taking part in the life of the community on an equal level with others due to physical and social barriers’ [[17],p.27]. Sen’s conceptualisation draws attention to how environments ridden with barriers restrict the functionings attainable by disabled people – that is, exacerbate poverty.

Both functional limitation and environmental barriers place disabled people in poor communities at a further remove from the means to exercise capabilities. To Sen, capability is not the presence of ability, instead, it is a practical opportunity, while functioning refers to an individual’s actual achievement of being or doing [18]. In this framework, disability appears as a deprivation in terms of capabilities and functionings, emanating from the interaction between personal characteristics (such as age or impairment), available resources (such as assets and income), and the political, economic, social, and cultural environment [18]. Functionings are a subset of capabilities – those the individual is able to pursue. The cost of achieving a certain capability will vary with different environments. For example, for people with mobility impairments, the achievement of mobility will vary enormously, depending on the accessibility of the built environment, and availability and cost of assistive devices [18].

Importantly, Sen’s model, when applied to disability, can reflect poverty where a traditional model – focusing purely on income and consumption – sees none. To illustrate, it is possible for a disabled individual to have a large income and possess assets, yet have less chance to pursue her life objectives than a nondisabled person of far less financial means [5]. Sen chose to deliberately resist creating a rigid taxonomy of personal and environmental factors and commodities, instead encouraging local, pluralistic application of his model. This allows for the taxonomy of needs to be populated in local idiom [5].

For our purposes, the work of Sen and others [18–20] sets a tone for research into poverty and disability which embraces complexity. Not only are the effects of disability on family well-being often subtle, but extreme poverty in one area of functioning may exist side-by-side with abundance in another. Picking up on these themes, Zaidi and Burchardt [21] show that disabled people may have a lower standard of living than nondisabled people with the same income, due to their differing needs. These needs encompass both disability-specific items, and greater amounts of general household commodities [15]. Households with disabled members may need to spend more on these items, diminishing the means available for resources which would raise the general living standard in the home [21]. In sum, work in this area directs attention toward the multi-dimensional ways in which poverty and disability intersect and overlap.

It has been an ideological position in disability studies to strategically emphasise the structural realities of poverty, in order to overcome the ‘medicalising’ view that impairment-based functional limitations are the most influential factor in the well-being of disabled people and their families [22,23]. This position is not one which we seek to perpetuate here. Writing about the significance of poverty does not mean supporting a view which construes functional limitations as of little or no consequence. In addition, attempts to clearly separate out the effects on families of poverty and disability are misleading – families do not experience hardship in terms of discrete difficulties. Instead, these adversities appear to be mutually constitutive.

## Methods

The authors conducted a selective review of the research record concerned with families with disabled children living in LMICs. Papers were then discussed and critiqued, and the findings supplemented by an additional purposive literature search.

### Procedure

We searched for English language peer-reviewed journal articles containing a combination of search terms pertaining to *disability* were used (e.g., disab*, handicap*, bifida*, sclerosis*), *family* (e.g., parent*, family*), *poverty* (e.g., poverty*, poor*), and *low- and middle-income countries* (e.g., Global South*, low-resource*, developing countries*) within several scientific databases.

The authors sorted all emerging articles by topic. When it became apparent that intervention literature, family quality of life (FQOL), and work examining the cultural facets of disability experience were the most prominent categories of literature, additional searches were conducted with these terms.

Each paper was examined, and given a quality rating based on, (1) the clarity of explanation of methods/replicability; (2) the sample size; and (3) the depth of analysis/discussion. These ratings guided our prioritisation of each piece in the results (with insights gleaned from a paper rated 3 holding more weight than one rated 1). Literature concerning each of these bodies of work was then consolidated; the review articles from each were précised, and augmented with individual papers from their field which contributed novel insights not afforded by the review. The authors then critically analysed and discussed the three bodies of research, comparing and contrasting them, and thinking through the implications of the contributions and elisions of each. This process informed the discussion which follows, and the recommendations stemming from it.

## Results

The three types of research which emerged from the review were categorised as:

1. Research examining FQOL of families with a disabled child.
2. Studies documenting the nature and efficacy of interventions for supporting families with a disabled child in LMICs.
3. Studies emphasising how local cultural norms mediate the experience of disability for families, and their participation in supportive interventions.

Our search identified 85 articles which were either research studies or theoretical pieces, and 10 review articles. Twenty-three pertained to the cultural aspects of family functioning in the context of disability (one review), ten concerned Family Quality of Life (FQOL) (one review), and eight were intervention articles (two reviews). These are listed in Table A1 (see Appendix). We also found 30 papers pertaining to the relationship between disability and poverty (six reviews). These latter papers form the background to this paper, and are not discussed as part of the results.

## Disability, poverty, and FQOL

FQOL refers to a state of being in which family members: (1) have their needs met; (2) enjoy their lives together; and (3) have opportunities to pursue goals they consider meaningful, in each of ten life domains [24]. The key review of literature in the area of FQOL where the families include a disabled child, by Park, Turnbull, and Turnbull [25], is over a decade old. We begin by drawing together salient aspects of this work, and then update the account with recent publications.

Park, Turnbull, and Turnbull [25] review available studies on the impact of poverty on FQOL for families with a disabled child, noting that it is now clear that the impact of poverty on a range of developmental outcomes for all children is substantial. The research reviewed by Park, Turnbull, and Turnbull [25] emerges exclusively from the USA; thus, its application to Global South contexts must be cautious. Like Park, Turnbull, and Turnbull [25], we aimed to limit our review to the effects of poverty on FQOL in families with a disabled member. Still, this literature overwhelmingly reflects research performed in Global North contexts, which is, for our purposes, obviously a difficulty. Our reasoning has been to examine only FQOL studies from the Global North which include the variable of poverty, while remaining cognisant that the lived nature of poverty in the Global North differs from that in the Global South.

The review of Park, Turnbull, and Turnbull [25] demonstrates a host of ‘poverty effects’ which apply to all families, but in some instances are more severe in families with a disabled member. For instance, the impact of hunger resulting from poverty on general well-being is self-evident, but with heightened risks for disabled children [25].

Four additional articles, published since this review paper, deal with FQOL amongst families with a disabled child in contexts of poverty in the Global South. Yagmurlu, Yavuz, and Sen [26] found that the well-being of mothers in a disadvantaged community in Turkey was closely associated with economic and social factors and stress, not the child’s disability. Similarly, Meral, Cavkaytar, Turnbull et al. [27], in a study of FQOL amongst Turkish families with a disabled child, found that perceptions of FQOL were lowest in the physical/material well-being domain. These findings have also been replicated in Catalonia [28], where employment status, and family income, particularly in households with children under 18 years of age, predicted lower FQOL scores. Similar conclusions were reached by Aldersey, Francis, Haines et al. [29], who explored the appropriateness of adapting FQOL measurement tools for use in a Congolese context. Participants identified poverty as a crucial underlying factor in FQOL in the DRC (Democratic Republic of Congo).

These studies suggest that for families with a disabled child living in a resource-scarce community, it is poverty, rather than the child’s functional limitations, which cause the most immediate subjective distress. One may assume, though, that this poverty is exacerbated by disability as part of the ‘vicious cycle’.

## Supportive interventions in LMICs

Families typically constitute the primary source of capabilities for responding to the challenges associated with disability [30]. In the Global South, availability of personal assistance services is far less likely than in the Global North. This amplifies the burden of care which falls on families, highlighting the need to focus intervention efforts on the family unit. A dearth of suitably qualified health practitioners means that primary caregivers have often been trained to lead disability-related interventions for children and families [31]. Indeed, the lone review which emerged concerning interventions, examined parent-led interventions for children with intellectual disabilities.

Einfeld, Stancliffe, Gray et al. [32] identified interventions for children with intellectual disabilities, deliverable by families, which have been implemented in LMICs. The focus of the review is on examining the quality of the evidence supporting such interventions, rather than on making broad recommendations about intervention itself. Conclusions, consequently, pertain more to the need for better quality of evidence, than to types of intervention which might be useful in LMICs.

It is necessary – given the specific focus of their paper – to add a more inclusive overview of the family-disability intervention literature. However, some of the observations which we can make regarding Einfeld, Stancliffe, Gray et al.’s [32] parent-led interventions for families with a disabled child, hold for other types of intervention too.

For instance, interventions for families with a disabled member work within the structural limitations of economic context, but do not problematise or attempt to address these (below is a notable exception). While passing reference is made to the need for cultural sensitivity in the delivery of intervention procedures, no meaningful attention is paid to the ways in which all aspects of context, including culture, may determine intervention priorities.

Interventions are often focussed on specific areas of perceived difficulty: for instance, child behavioural management. This sidelines the fact that poverty may underlie the most urgent concerns which a family faces. These interventions are focussed on helping families to cope with disability, not the multidimensional poverty which disability, in interaction with the environment, may engender.

However, there is an emerging branch of intervention which works directly to try to change the families’ structural circumstances. Cash benefits have become more widely utilized in many types of intervention in recent years. Medeiros, Diniz, and Squinca [33] report on the Continuous Cash Benefit Programme (BPC, which stands for Benefício de Prestação Continuada in Portuguese), an unconditional cash transfer to the elderly or to extremely poor individuals with disabilities in Brazil. A strength of this intervention is the fact that it targets individuals and not families, and by so doing might still help individuals whose families benefit from other assistance, leaving such an individual above the poverty threshold for assistance. In other words, it takes into account the findings of Braithwaite and Mont [15] that poverty, in LMIC families with a disabled child, is not necessarily equally distributed between all members of the household, as well as the finding that individual assistance is required to get families of a disabled child closer to the population baseline in terms of functionings, than other families on social support. This intervention gives an extra nudge to the disabled members of families in recognition of their greater relative distance from Sen’s functionings than non-disabled persons, and allows for the local determination of where and how this money is spent.

On the other hand, this intervention does not take into account the loss of income to caregivers and families as a whole. As Braithwaite and Mont [15] suggest, when parents must care for a disabled child, this alters their production function (for example, taking time out of one’s day for care when one could be working), and so parents will be more impoverished than in a family without a disabled member, even though the disability is not their own.

## Culture and contextual specificity

In this concluding section, we review the literature concerning the intersection of culture, disability and the family. Culture, in the words of Gurung [34], encompasses ‘a dynamic yet stable set of goals, beliefs, and attitudes shared by a group of people’ (p.448). Culture frames our worldview and helps us make sense of what we know [35].

A family’s cultural frame of reference must be viewed against the broader socio-economic and geopolitical context. Although the paper does not report on families specifically living in poverty, nor LMICs, the insights which it afforded to our discussion of culture here warranted its inclusion.

The lone review concerning the intersection of disability and the family, and culture, was conducted in 2012 by Ravindran and Myers [35]. It examined papers concerning the cultural influences on parental perceptions of autism. Cultural influences affect fundamental aspects of the treatment process including whether or not, and in what ways, families seek help, what interventions might be appropriate, which resources are available, and how professional-family relationships play out. The writers describe how a broad cultural view can help researchers understand treatments and treatment delivery systems [35]. Further, the paper highlights the fact that the best-practice ways of intervening with families must be assessed for their suitability in other cultural contexts [35].

Despite its considerable contribution, the review of Ravindran and Myers [35] does not attend to how poverty, as a third variable in the disability-culture nexus, might influence these priorities too. Interventions which thoroughly acknowledge the context of the child would take into consideration the strengths and limitations of the child, the family, and their broader socio-economic and cultural environment, rather than cultural mores alone. Therefore, we have supplemented the insights of this paper with those afforded by articles addressing cultural models and disability – including a broader range of disabilities – in the Global South since 2012.

In 2012, Cohen [36] examined the possibilities for comprehensive care for children with intellectual disabilities amongst low-income Latino families. Cohen [36] notes that, when community services and supports are in place, the strains of caring for a disabled child in LMICs can be ameliorated. He examines the ways in which a cultural lens, drawing on traditional mechanisms of community care, can allow helping professionals and families to mobilise care for disabled children.

Fellin, Desmarais, and Lindsay [37], rather than focussing on the cultural models of families, turn our attention to clinicians’ experiences of delivering collaborative, culturally competent services to immigrant families raising a child with a physical disability. These authors report that clinicians either remove, or create, barriers to care for immigrant families in different ways. Their findings suggest that there is a need for more ‘institutional support for collaborative, culturally competent care to immigrant families raising a child with physical disability’ [36,p.1961].

While this recommendation is surely accurate, its applicability to contexts with a dearth of such institutional support is questionable. Further, in some Global South contexts, particularly post-colonial ones, the issue of providing culturally competent care is not a matter of accommodating a minority of immigrants: instead, the challenge is to develop a health system, often populated by a minority of privileged persons working within a western care paradigm, into one which is: (1) representative of the broader population, and their systems of meaning, and (2) which responds in a socio-economically and culturally appropriate manner to the needs of families.

Attending to these factors, Njelesani, Leckie, Drummon et al. [38] examined parental perceptions of barriers to physical activity in disabled children living in Trinidad and Tobago. Indeed, what emerged from their study was that it was a combination of the families’ individual, culturally-mediated priorities for their child, in combination with the wider socio-economic environment in which they lived, which determined the child’s degree of participation.

## Discussion

When examined in conjunction, the research clusters discussed here contribute to an enriched, nuanced image of families with disabled children in the Global South. By contrast, when the research strands are considered in isolation, the record shows as a web of gaps, with each strand speaking at cross-purposes, despite their commonalities, and failing to learn from the lessons of the other. Below follows a summary of the unique contributions made by each:

### Family quality of life

Before turning to the literature, a superordinate critique of the FQOL framework must be made. The notion that the states which embody satisfactory quality of life for a family in Kansas (where the model originated), are the same as the states which embody satisfactory quality of life in, say, Rwanda, is problematic. Equally problematic is the premise, on which FQOL measures rest, that the quality of life of an individual family can be assessed against a set of common criteria of ‘things which are good for families’.

Still, research on FQOL makes a twofold contribution to our thinking on the disability-family nexus, in the context of the Global South. Firstly, although this is a simplification, it points to poverty – or lack – as a primary source of family distress. Secondly, it sets our focus squarely on the families in question, their lived experience, and their expertise in navigating their circumstances.

However, if we examine the contributions of FQOL research in the context of the poverty-disability intersection, we see that it misses certain opportunities. Specifically, the work of Meral, Cavkaytar, Turnbull et al. [27] and Giné, Gràcia, Vilaseca et al. [28] shows us that in the Global South, economic factors may be of more immediate import to families’ quality of life than in the Global North, and so our interventions, and measurement of FQOL, should take this into account. In addition, although these papers point to poverty as impinging on FQOL for families with a disabled child, the instruments used, and findings yielded, are not nuanced enough to shed light on how this factor and disability interact.

### Intervention research

The main contributions of intervention research from the Global South are, firstly, its highlighting of the family as the unit of focus, and secondly, its re-emphasising of the numerous contextual factors which must be considered when working with Global South families. But in relation to the other work discussed here, omissions appear. As noted, when we consider the types of interventions discussed by Einfeld, Stancliffe, Gray et al. [32] in relation to work on FQOL, we see consideration of what is most impinging on FQOL amongst LMICs families of a disabled child – for instance, Meral, Cavkaytar, Turnbull et al.’s [27] finding that it is lack of resources, and Yagmurlu, Yavuz, and Sen’s [26] that it is lack of emotional support. This could inform intervention foci, which casts a favourable light on cash-transfer type programmes. Note though, that if we see cash-transfer type programmes within the context of the disability-family-poverty work, we see that, as the deprivation engendered by disability might be family-wide, assistance should possibly be twofold: a disability grant for the child, as well as for the parents, whose loss of income due to caregiving responsibilities could have knock-on effects for the other household members.

### Culturally oriented research

Finally, the work on culture draws attention to locally-determined priorities of families with a disabled child in the Global South. This has implications not only for which interventions are suitable for such families, but also for which needs will be seen by these families as most pressing.

Nevertheless, it too has shortcomings, especially when considered in light of the accumulated evidence discussed thus far. If we consider the work of Ravindran and Myers [35] in relation to the FQOL research, we gain additional insights into how a consideration of culture in relation to families of children with disabilities might be enriched. Ravindran and Myers’ [35] work, due to its focus, rather than an oversight, does not consider how cultural models might impact on FQOL in a family with a disabled child: for instance, it might be more ‘detrimental’ to FQOL to have a hearing-impaired child if having a hearing-impaired child is seen as a curse or bad Karma, and less so if the impairment is understood in biomedical terms. In combination, then, an assessment of family priorities highlighted by a consideration of culture and socio-economic status in the Global South, would also likely be enhanced by paying attention to how these same factors would impact on FQOL.

When we recall the interventions reviewed by Einfeld, Stancliffe, Gray et al. [32], we see how work on cultural models could inform interventions, drawing attention to the contextually- and culturally-determined priorities of families in a particular place and time (recall that Sen makes provision for locally-determined prioritisation of certain functionings over others). Considering culture in relation to families’ ways of thinking about health, illness, and disability reminds us that treatment approaches developed in the Global North cannot simply be generalised to persons in the Global South [39], a principle which is demonstrated in the implementation of manualised treatments. Finally, in relation to FQOL, culture will directly determine what experiences embody FQOL in a given context.

## Conclusion

We concur with Braithwaite and Mont [15] in recommending Sen’s poverty framework as a tool for thinking about families with a disabled child in the Global South. Disability shows up how important cultural assessments of value and flourishing are in understanding poverty, because so much of the lived impoverishing implications of disability are subtle and context specific, as well as mediated by cultural significations.

Further, our work here suggests, current prominent bodies of research concerning families with a disabled child in the Global South each bear insights into one or more aspects of their target focus, but neglects others. When stitched together, the combined understanding of disability in families which we have presented offers a troubling of simplistic conceptions of poverty, income, and consumption. The complexity of the predicaments of Global South families with a disabled child needs to be foregrounded in future work, rather than underestimated through narrow application of an individual framework.

## Paper context

Research approaches to the impact on Global South families living in poverty of caring for a child with disabilities are limited and disparate. We divide these into three categories, pertaining to family quality of life, interventions, and cultural meanings. Using Sen’s ‘capabilities’ model of poverty, this paper distils key insights for future research from this work, while critiquing theory and method for its limited applicability to LMI countries, and neglect of the family-disability-poverty nexus.

## Appendix

**Table A1.** Summary of publications reviewed.

Reference		Quality
**Culture**		
1	Powell SE, Bauer JW. Resource use of rural low-income families caring for children with disabilities. J Child Poverty. 2010;16(1):67–83.	n = 26 families qualitative and quantitative overall quality rating^a^ 3
2	Rao S. ‘A little inconvenience’: perspectives of Bengali families of children with disabilities on labelling and inclusion. Disabil Soc. 2001;16(4):531–548.	n = 8 qualitative and observational overall quality rating^a^ 3
3	Yousafzai AK, Farrukh Z, Khan K. A source of strength and empowerment? An exploration of the influence of disabled children on the lives of their mothers in Karachi, Pakistan. Disabil Rehabil. 2011;33(12):989–998.	n = 18 qualitative overall quality rating^a^ 2
4	Cohen SR. Advocacy for the ‘Abandonados’: harnessing cultural beliefs for Latino families and their children with intellectual disabilities. J Policy Pract Intellect Disabil. 2013;10(1):71–78.	theoretical/discussion paper
5	Harry B. Collaboration with culturally and linguistically diverse families: Ideal versus reality. Except Child. 2008;74(3):372–388.	theoretical/discussion paper
6	Xu Y. Empowering culturally diverse families of young children with disabilities: The double ABCX model. Early Child Educ J. 2007;34(6):431–437.	theoretical/discussion paper
7	Foley D, Chowdhury J. Poverty, social exclusion and the politics of disability: Care as a social good and the expenditure of social capital in Chuadanga, Bangladesh. Soc Policy Adm. 2007;41(4):372–385.	n = 10 qualitative overall quality rating^a^ 2
8	Porterfield SL, McBride TD. The effect of poverty and caregiver education on perceived need and access to health services among children with special health care needs. Am J Public Health. 2007;97(2):323–329.	n = 38 866 quantitative overall quality rating^a^ 3
9	Fellin M, Desmarais C, Lindsay, S. An examination of clinicians’ experiences of collaborative culturally competent service delivery to immigrant families raising a child with a physical disability. Disabil Rehabil. 2015;37(21):1961–1969.	n = 43 qualitative overall quality rating^a^ 3
10	Gona JK, Newton CR, Hartley S, et al. A home-based intervention using augmentative and alternative communication (AAC) techniques in rural Kenya: what are the caregivers’ experiences? Child Care Health Dev. 2014;40(1):29–41.	n = 9 quantitative overall quality rating^a^ 2
11	Danseco ER. Parental beliefs on childhood disability: Insights on culture, child development and intervention. Intl J Disabil Dev Educ. 1997;44(1):41–52.	theoretical/discussion paper
12	Harry B. Making sense of disability: Low-income, Puerto Rican parents’ theories of the problem. Except Child. 1992;59(1):27–40.	n = 12 qualitative overall quality rating^a^ 3
13	Maloni PK, Despres ER, Habbous J, et al. Perceptions of disability among mothers of children with disability in Bangladesh: Implications for rehabilitation service delivery. Disabil Rehabil. 2010;32(10):845–854.	n = 11 qualitative overall quality rating^a^ 3
14	Mutua NK, Miller JW, Mwavita M. Resource utilization by children with developmental disabilities in Kenya: discrepancy analysis of parents’ expectation-to-importance appraisals. Res Dev Disabil. 2002;23(3):191–201.	n = 351 quantitative overall quality rating^a^ 3
15	Pan L, Ye J. Family care of people with intellectual disability in rural China: A magnified responsibility. J Appl Res Intellect Disabil. 2015;28(4):352–366.	n = 2 qualitative overall quality rating^a^ 1
16	Ravindran N, Myers BJ. Cultural influences on perceptions of health, illness, and disability: A review and focus on autism. J Child Fam Stud. 2012;21(2):311–319.	Review
17	Harry B. Trends and issues in serving culturally diverse families of children with disabilities. J Spec Educ. 2002;36(3):132–140.	theoretical/discussion paper
18	Njelesani J, Leckie K, Drummond J, et al. Parental perceptions of barriers to physical activity in children with developmental disabilities living in Trinidad and Tobago. Disabil Rehabil. 2015;37(4):290–295.	n = 9 qualitative overall quality rating^a^ 2
19	Rueda R, Monzo L, Shapiro J, et al. Cultural models of transition: Latina mothers of young adults with developmental disabilities. Except Child. 2005;71(4):401–414.	n = 16 qualitative overall quality rating^a^ 3
20	Blacher J, McIntyre LL. Syndrome specificity and behavioural disorders in young adults with intellectual disability: Cultural differences in family impact. J Intellect Disabil Res. 2006;50(3):184–198.	n = 282 quantitative overall quality rating^a^ 3
21	Skinner D, Rodriguez P, Bailey DB Jr. Qualitative analysis of Latino parents’ religious interpretations of their child’s disability. J Early Interv. 1999;22(4):271–285.	n = 250 qualitative overall quality rating^a^ 2
22	Raghavan R, Pawson N, Small N. Family carers’ perspectives on post-school transition of young people with intellectual disabilities with special reference to ethnicity. J Intellect Disabil Res. 2013;57(10):936–946.	n = 43 qualitative overall quality rating^a^ 3
23	Cohen SR, Holloway SD, Domínguez-Pareto I, et al. Receiving or believing in family support? Contributors to the life quality of Latino and non-Latino families of children with intellectual disability. J Intellect Disabil Res. 2014;58(4):333–345.	n = 145 quantitative overall quality rating^a^ 2
**Interventions**		
1	Patel S, Alavi Y, Lindfield R, et al. The impact of rehabilitative services in the lives of adults and children with disabilities, in low-income and middle-income countries: an assessment of the quality of the evidence. Disabil Rehabil. 2013;35(9):703–712.	Review
2	Medeiros M, Diniz D, Squinca F. Cash benefits to disabled persons in Brazil: an analysis of BPC-continuous cash benefit programme. Textos para Discussão (IPEA). 2015;1184.	theoretical/discussion paper
3	Einfeld SL, Stancliffe RJ, Gray KM, et al. Interventions provided by parents for children with intellectual disabilities in low and middle income countries. J Appl Res Intellect Disabil. 2012;25(2):135–142.	Review
4	Narayanan HS, Girimaji SR, Gandhi DH, et al. Brief in-patient family intervention in mental retardation. Indian J Psychiatry. 1988;30(3):275.	n = 106 quantitative overall quality rating^a^ 3
5	McConachie H, Huq S, Munir S, et al. Difficulties for mothers in using an early intervention service for children with cerebral palsy in Bangladesh. Child Care Health Dev. 2001;27(1):1–12.	n = 47 quantitative overall quality rating^a^ 3
6	McConkey R, Mariga L, Braadland N, et al. Parents as trainers about disability in low income countries. Intl J Disabil Dev Educ. 2000;47(3):309–317.	n = 21 qualitative overall quality rating^a^ 2
7	Russell PS, al John JK, Lakshmanan JL. Family intervention for intellectually disabled children. Randomised controlled trial. Br J Psychiatry. 1999;174(3):254–258.	n = 57 quantitative overall quality rating^a^ 3
8	Mitra S. Disability and social safety nets in developing countries; 2005. Available at http://documents.worldbank.org/curated/en/443641468140637787/pdf/327400rev.pdf	theoretical/discussion paper
Family quality of life (**FQOL)**		
1	Park J, Turnbull AP, Turnbull HR III. Impacts of poverty on quality of life in families of children with disabilities. Except Child. 2002;68(2):151–170.	Review
2	Meral BF, Cavkaytar A, Turnbull AP, et al. Family quality of life of Turkish families who have children with intellectual disabilities and autism. Res Pract Persons Severe Disabil. 2013;38(4):233–246.	n = 3009 quantitative overall quality rating^a^ 3
3	Giné C, Gràcia M, Vilaseca R, et al. Family quality of life for people with intellectual disabilities in Catalonia. J Policy Pract Intellect Disabil. 2015;12(4):244–254.	n = 144 quantitative overall quality rating^a^ 3
4	Wang M, Turnbull AP, Summers JA, et al. Severity of disability and income as predictors of parents’ satisfaction with their family quality of life during early childhood years. Res Pract Persons Severe Disabil. 2004;29(2):82–94.	n = 364 quantitative overall quality rating^a^ 2
5	Werner S, Edwards M, Baum N, et al. Family quality of life among families with a member who has an intellectual disability: an exploratory examination of key domains and dimensions of the revised FQOL Survey. J Intellect Disabil Res. 2009;53(6):501–511.	n = 35 quantitative overall quality rating^a^ 2
6	Gardiner EC. Quality of life in families of children with autism spectrum disorder: considerations of risk and resilience [doctoral dissertation: Arts & Social Sciences]; 2014.	n = 84 quantitative and qualitative overall quality rating^a^ 3
7	Yagmurlu B, Yavuz HM, Sen H. Well-being of mothers of children with orthopedic disabilities in a disadvantaged context: findings from Turkey. J Child Fam Stud. 2015;24(4):948–956.	n = 105 quantitative overall quality rating^a^ 3
8	Schertz M, Karni-Visel Y, Tamir A, et al. Family quality of life among families with a child who has a severe neurodevelopmental disability: Impact of family and child socio-demographic factors. Res Dev Disabil. 2016;53:95–106.	n = 70 quantitative overall quality rating^a^ 3
9	Dardas LA, Ahmad MM. Quality of life among parents of children with autistic disorder: A sample from the Arab world. Res Dev Disabil. 2014;35(2):278–287.	n = 184 quantitative overall quality rating^a^ 2
10	Aldersey HM, Francis GL, Haines SJ, et al. Family quality of life in the Democratic Republic of the Congo. J Policy Pract Intellect Disabil. 2017;14(1):78–86.	n = 21 qualitative overall quality rating^a^ 2

^a^The quality assessment ranks each paper out of 3 according to: (1) clarity of explanation of methods/replicability; (2) sample size; and (3) depth of analysis/discussion.
